# Research on the Constitutive Model of PTFE/Al/Si Reactive Material

**DOI:** 10.3390/polym14071358

**Published:** 2022-03-27

**Authors:** Liangliang Ding, Xiaoxiao Cui, Wenhui Tang, Xue Zhong, Yuli Zhao, Yongzheng Huang, Peng Shi, Xiaoguang Xue

**Affiliations:** 1 Beijing Institute of Tracing Telecommunication Technology, Beijing 100028, China; dingliangliang14@nudt.edu.cn (L.D.); tatsuyahugh@163.com (X.C.); way9092@163.com (Y.Z.); yongzheng0531@126.com (Y.H.); lyship1970@126.com (P.S.); 2 College of Liberal Arts and Sciences, National University of Defense Technology, Changsha 410073, China; 3 China Aerodynamics Research and Development Center, Mianyang 621000, China; zhongxue@cardc.cn

**Keywords:** reactive material, PTFE/Al/Si, quasi-static mechanical properties, dynamic mechanical properties, SHPB, Johnson-Cook constitutive model, strain hardening effect, strain rate hardening effect, thermal softening effect

## Abstract

As a new type of energetic material, reactive materials are widely used at present; in particular, the metal/polymer mixtures type reactive materials show great advantages in engineering applications. This type of reactive material has good mechanical properties, and its overall performance is insensitive and high-energy under external impact loading. After a large number of previous studies, our team found that the energy release characteristics of PTFE/Al/Si reactive material are prominent. In order to master the mechanical properties of PTFE/Al/Si reactive materials, the quasi-static mechanical properties and dynamic mechanical properties were obtained by carrying out a quasi-static compression test and a dynamic SHPB test in this paper. Based on the experimental data, a Johnson-Cook constitutive model of PTFE/Al/Si reactive material considering strain hardening effect, strain rate hardening effect and thermal softening effect was constructed. The relevant research results will be used to guide future research on the reaction mechanism of PTFE/Al/Si reactive materials, in order to promote the engineering application of PTFE/Al/Si reactive materials.

## 1. Introduction

Reactive materials are new energetic materials, which were first discovered in the 1970s and then widely studied and applied [1]. Types of reactive materials mainly include the following: thermite (e.g., Al/CuO), metal/polymer mixtures (e.g., Al/PTFE), intermetallic compounds (e.g., Al/Ni), metastable intermolecular compounds (e.g., Al/MoO_3_), etc. In recent years, metal/polymer mixture type reactive materials have received full attention, especially reactive materials based on fluoropolymers and active metals, which show great advantages in engineering applications [2,3,4]. Taking PTFE/Al (73.5%/26.5%) reactive material as an example, its overall performance is insensitive and high-energy under external impact loading conditions, and its unit mass energy and unit volume energy can reach 3.5 times and 5 times that of TNT explosives, respectively [5].

For PTFE-based reactive materials, many scholars have carried out a large number of mechanical properties tests and energy release characteristics. Cai [6,7,8] carried out a large number of mechanical properties tests on PTFE/Al and PTFE/Al/W reactive materials, including a quasi-static compression test, drop hammer impact test and dynamic SHPB test. Zhang [9,10] studied the effect of the composition ratio of PTFE/Al/W reactive material on the compression performance of the material; the test results show that the three PTFE/Al/W reactive materials with different ratios show an obvious strain rate effect. Casem [11] studied the mechanical properties of PTFE/Al (26.5:73.5) reactive material at different temperatures through a quasi-static compression test and a dynamic SHPB test in 2008 and found that the material showed an obvious strain rate hardening effect and a strain hardening effect. Wu [12,13] studied the effect of Al particle size on the mechanical properties of PTFE/Al reactive materials through a quasi-static compression test and a drop hammer impact test and analyzed the mechanical properties and reaction characteristics of PTFE/Al reactive materials after adding an Ni component. Ames [14] and Mock [15,16] designed multiple types of impact loading tests, such as direct impact, indirect impact and two-step impact, and deeply studied the impact reaction behavior and initiation mechanism of PTFE/Al reactive material. According to the test results, it is speculated that the impact initiation of reactive material is likely to be related to crack propagation characteristics, fracture surface energy and hole collapse. Li [17] carried out dynamic SHPB compression tests on PTFE/Al specimens prepared under different forming pressures to study the dynamic mechanical behavior and impact ignition characteristics of PTFE/Al reactive materials. Ren [18] carried out the dynamic energy release test of PTFE/Al/W reactive materials with different composition ratios. By drawing on the energy release test method of explosives (i.e., the characteristic drop height method), the strain rate threshold of reactive materials with different composition ratios was obtained. Zhou [19] carried out a series of tests on the energy release capacity of PTFE/Al/W reactive material in a quasi-closed vessel and obtained the pressure-time curves of the reactive material inside the vessel after chemical reaction under different impact velocities, thus deriving the chemical reaction degree of the reactive material corresponding to each working condition. Ge [20] carried out a series of light gas gun tests on PTFE/Al reactive materials, studying the impact reaction thresholds and impact induction mechanism of PTFE/Al reactive materials under different impact loading conditions.

Our research team has also carried out a lot of work on PTFE-based reactive materials and has obtained some research results. Zhou [21] studied the mechanical properties and impact-induced reaction characteristics of PTFE/Al/CuO reactive materials through a quasi-static compression test, a dynamic SHPB test and a drop hammer impact test, and also considered the effects of particle size and component distribution ratio of each matrix. Ding [22] studied the impact energy release characteristics of PTFE/Al/CuO reactive materials with a self-designed energy release testing device, and the effects of particle size, the ratio of PTFE/Al and Al/CuO materials and sintering on the energy release ability of the reactive materials were investigated. Zou [23] studied the ignition height and reaction flame temperature of PTFE/Al/Si/CuO reactive materials with different mass ratios of PTFE/Si by a drop hammer impact test. Ran [24] quantitatively and qualitatively analyzed the energy release ability of six kinds of reactive materials formulations (PTFE/Al, PTFE/B, PTFE/Si, PTFE/Al/B, PTFE/Al/Si, and PTFE/Al/CuO) by using the drop weight system and a self-designed energy release testing device, and the results showed that the PTFE/Al/Si formulation had the best energy release ability. Based on the above research results, our team has carried out a series of research works focusing on PTFE/Al/Si reactive materials. This paper will mainly introduce the quasi-static mechanical property test and dynamic mechanical property test of PTFE/Al/Si reactive materials, and will build a constitutive model which can characterize PTFE/Al/Si reactive materials based on the obtained test data. The relevant research results will be used to guide future research on the reaction mechanism of PTFE/Al/Si reactive materials, in order to promote the engineering application of PTFE/Al/Si reactive materials.

## 2. Quasi-Static Mechanical Properties Test of PTFE/Al/Si Reactive Material

Mechanical properties tests are the most direct and effective test method to study the mechanical behavior of materials, which mainly include a quasi-static mechanical properties test and a dynamic mechanical properties test. For quasi-static mechanical properties test, the strain rate is usually less than 1 s^−1^; even after special modification design, it is not more than 100 s^−1^. The dynamic mechanical properties test usually corresponds to high strain rate, which ranges from 10^2^~10^4^ s^−1^. The mechanical properties of materials under quasi-static conditions are different from those under dynamic conditions, which is mainly reflected in the fact that the inertia effect of materials will increase significantly with the increase of loading strain rate. Therefore, in order to master the mechanical behavior of PTFE/Al/Si reactive material, it is necessary to carry out quasi-static and dynamic mechanical properties research. This section will carry out quasi-static mechanical properties research of PTFE/Al/Si reactive material, and its dynamic mechanical properties research will be carried out in the next section.

### 2.1. Basic Test Principle of Quasi-Static Compression Test

Since the reactive material studied in this paper is mainly based on PTFE, the quasi-static mechanical properties test of PTFE/Al/Si reactive material can refer to the test method of plastic compression properties. The entire quasi-static compression test needs to be carried out under standard environmental conditions (ambient temperature: 23 ± 2 °C, air relative humidity: (50 ± 15)%); the sample shape is cylinder and the sample size is ∅ 10 × 10 mm.

In the compression test, the relationship between the loading strain rate and the loading speed of the pressure testing machine is as follows:

(1) $$\dot{\varepsilon }=v/h_{s}$$

where $\dot{\varepsilon }$ is the loading strain rate of the testing machine punch (unit: s^−1^), *v* is the loading speed (unit: mm/s) and $h_{s}$ is the height of the specimen (unit: mm). The size of the quasi-static sample prepared in this paper is ∅ 10 × 10 mm, and the loading speed of the punch during the test is 0.01 mm/s. According to the Formula (1), the loading strain rate of the quasi-static test is 0.001 s^−1^.

According to the test principle, the engineering stress−strain relationship of the reactive material can be obtained from the quasi-static test. The expression of engineering stress and engineering strain is as follows:

(2) $$\sigma_{eng}=\frac{F}{A_{s}};\;\;\;\varepsilon_{eng}=\frac{\Delta h}{h_{s}}$$

The material is assumed to be incompressible, the volume of the sample remains unchanged during compression. Thus, the expression relation of the true stress *σ* and true strain *ε* can be obtained:

(3) $$\sigma =\frac{F}{A_{s}^{\prime }}=\frac{\sigma_{eng}A_{s}}{A_{s}^{\prime }};\;\;\;\varepsilon =\frac{\Delta h}{h_{s}^{\prime }}$$

(4) $$A_{s}\cdot h_{s}=A_{s}^{\prime }\cdot h_{s}^{\prime }$$

where *F* represents the pressure acting on the sample surface, *A_s_* and *h_s_* represent the initial cross-sectional area and initial height of the sample, respectively, *A_s_′* and *h_s_′* represent the cross-sectional area and height of the sample at any time, respectively, and ∆*h* represents the compression height of the sample. Therefore, according to the Formulas (2)–(4), the relationship between true stress−strain (σ−ε) and engineering stress−strain ($\sigma_{eng}-\varepsilon_{eng}$) can be obtained as follows:

(5) $$\sigma =\left(1-\varepsilon_{eng}\right)\sigma_{eng};\;\;\;\;\varepsilon =-\ln\left(1-\varepsilon_{eng}\right)$$

### 2.2. Sample Preparation and Test Results

**Figure 1:**
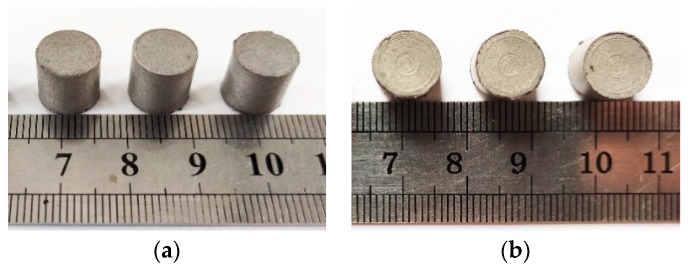
Physical samples of PTFE/Al/Si reactive material samples: (**a**) Front view; (**b**) Vertical view.

According to a large amount of research literature, the comprehensive mechanical properties of the sintered reactive materials are better than those of the unsintered reactive materials. Therefore, this paper only studies the mechanical properties of the sintered reactive materials. As mentioned above, the sample parameter used for the quasi-static compression test in this paper is ∅ 10 × 10 mm, and the loading strain rate in the quasi-static compression test is 0.001 s^−1^. The instrument used in the test is a hydraulic pressure testing machine (Jinan Tianchen Testing Machine Manufacturing Co., Ltd, Jinan, Shandong, China). In order to ensure the scientificity and consistency of the test results, three samples were prepared for the PTFE/Al/Si reactive material formula. The corresponding structural parameters are shown in Table 1, and the physical samples are shown in Figure 1.

**Figure 2:**
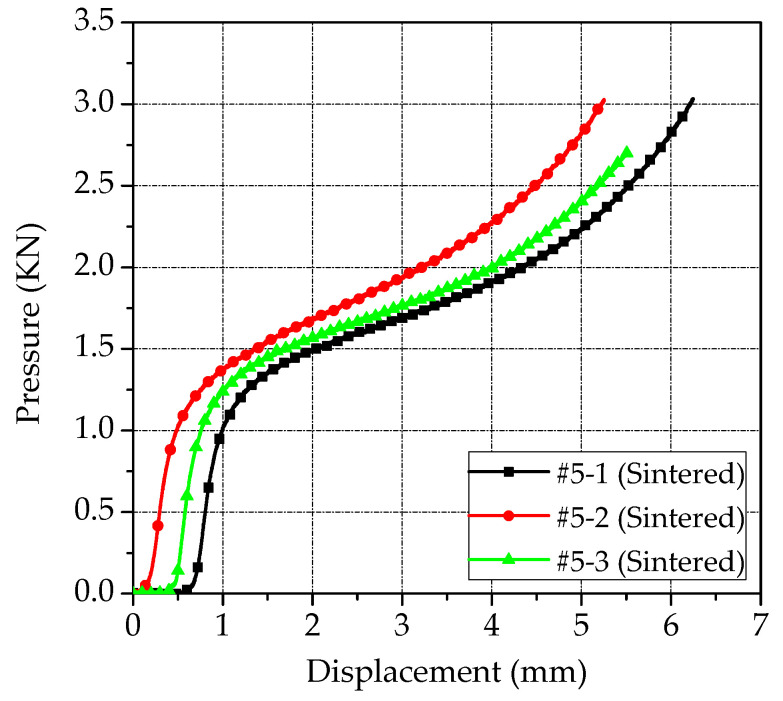
The pressure-displacement curves of the PTFE/Al/Si samples.

**Figure 3:**
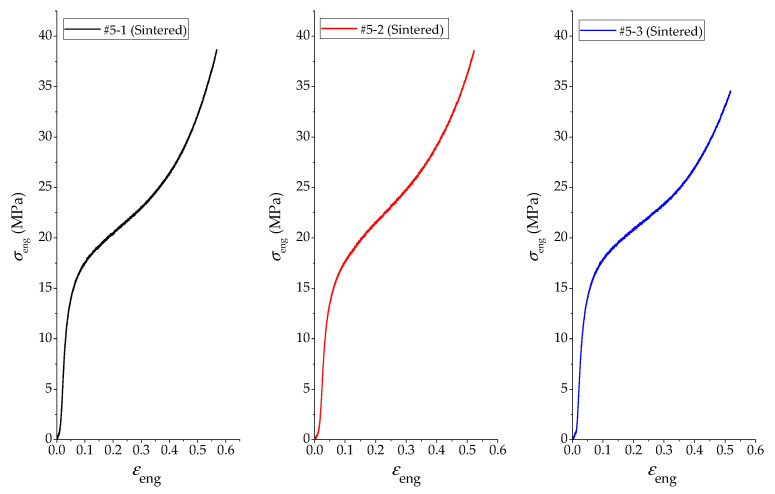
The engineering stress−strain curves of PTFE/Al/Si samples.

**Figure 4:**
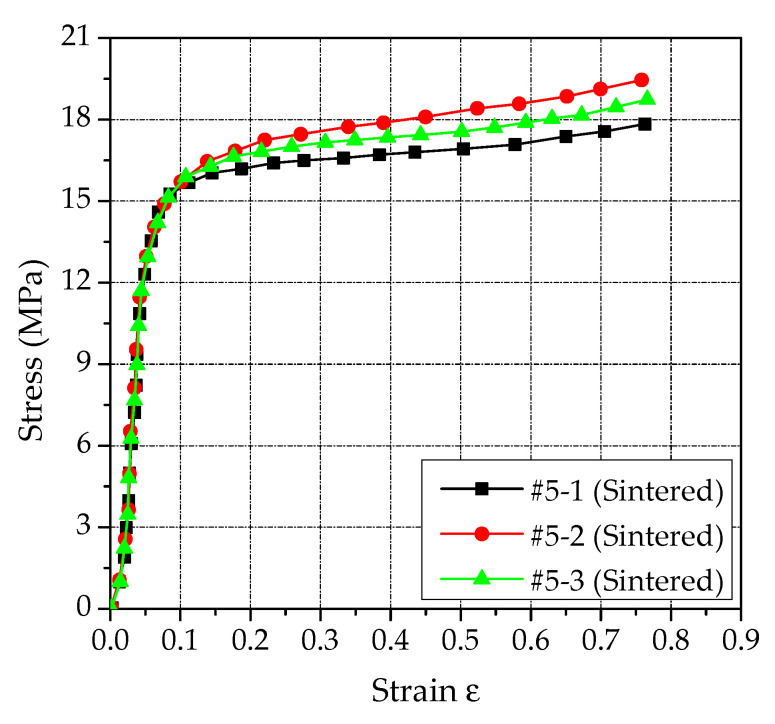
The real stress−strain curves of PTFE/Al/Si samples.

**Figure 5:**
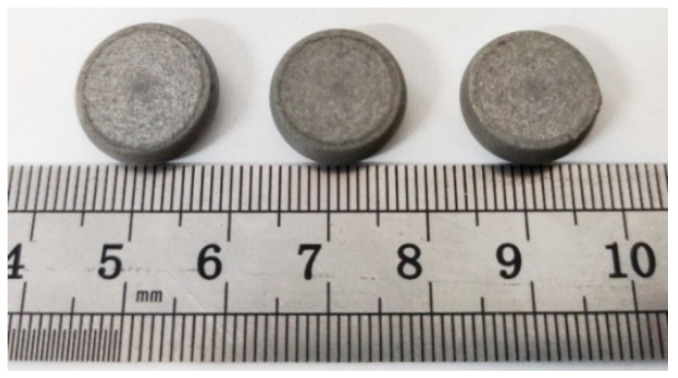
PTFE/Al/Si samples recovered from quasi-static compression test.

By processing the experiment data, the pressure-displacement curves of PTFE/Al/Si samples were obtained as shown in Figure 2. According to the Formulas (2) and (5), the engineering stress−strain curves and real stress−strain curves of PTFE/Al/Si samples were further obtained, as shown in Figure 3 and Figure 4, respectively. In addition, three PTFE/Al/Si samples recovered after the quasi-static compression test are shown in Figure 5.

It can be seen from Figure 2, Figure 3 and Figure 4 that the test curves of the three PTFE/Al/Si samples have good consistency. In addition, it can also be found that the stress−strain curve of the sintered PTFE/Al/Si sample under quasi-static loading shows very obvious elastic and plastic sections. In other words, the stress of the sintered PTFE/Al/Si sample in the elastic section shows a rapid upward trend with the increase of strain and then enters the plastic section. In the range of the plastic section, the stress increases slowly with the increase of strain, which reflects the strain hardening effect of the material. Generally speaking, the strain hardening effect of plastic section can be characterized by hardening modulus, which is the tangent slope of plastic section curve. According to the true stress−strain curve of PTFE/Al/Si sample shown in Figure 4, the main material parameters such as elastic modulus, hardening modulus, nominal yield strength and yield strain of the PTFE/Al/Si reactive material can be obtained, as shown in Table 2.

## 3. Dynamic Mechanical Properties Test of PTFE/Al/Si Reactive Material

In practical engineering applications, the strain rate effect and inertial effect of materials cannot be decoupled. However, the SHPB (Split Hopkinson Pressure Bars) test technology can solve this problem well and can effectively study the dynamic mechanical properties of materials under high strain rates. The core idea of SHPB test technology is that the stress wave propagating in the bar can have the dual functions of dynamic test and impact loading at the same time. Based on the stress wave information propagating in the bar, the stress−displacement−time (*σ*−*s*−*t*) relationship between the specimen end face and the bar can be solved, and the stress−strain (*σ*−*ε*) relationship of the specimen can be further obtained. Through the special design, the width of the loading wave is much larger than the thickness of the sample to be tested, so that the sample to be tested is in a local dynamic equilibrium during loading, and the influence of wave propagation is not considered in the deformation analysis of the sample to be tested. It can be seen that the SHPB test technology can separate the inertial effect and the strain rate effect of materials. For the sample to be tested, it is equivalent to the quasi-static test under high strain rate loading. For the bar, the dynamic response information of the sample to be tested is obtained by the inverse solution of the wave propagation information in the bar.

### 3.1. Basic Test Principle of Dynamic Compression Test

An SHPB pressure bar test device is mainly composed of an impact bar, an incident bar, a transmission bar, an absorption bar and other components. The device is developed by Kolsky [25] on the basis of the Hopkinson pressure bar experiment, and its basic principle is the elastic stress wave propagation theory based on two assumptions. The two core basic assumptions of SHPB pressure bar test technology are as follows:

(1) One-dimensional stress wave assumption. It is assumed that the wave propagating in the bar is a distortionless linear elastic wave, and both the incident bar and the transmission bar remain elastic during the impact. The diameter of the incident bar and transmission bar is much smaller than the wavelength of the incident wave, so the transverse dispersion effect of the stress wave propagation in the bar can be ignored, that is, there is only uniformly distributed axial stress in the bar.
(2) Uniformity assumption. When the incident wave is transmitted into the sample to be tested, the reflected wave and transmitted wave will be generated at the contact interface between the sample and the bar immediately, and then transmitted to the incident bar and the transmission bar. In the process of stress wave propagation, the stress wave in the sample will propagate back and forth between the two interfaces. If the size of the sample to be tested is small enough, the stress and strain distribution along the length direction in the sample will quickly reach homogenization.

Based on the above two basic assumptions, SHPB pressure bar test technology skillfully decouples the transverse effect and strain rate effect. In the test process, by controlling the impact velocity of the striker, the rise time of the generated incident pulse is short enough (usually less than 10 μs), which can realize the purpose of studying the dynamic response of the material under high strain rate loading.

**Figure 6:**
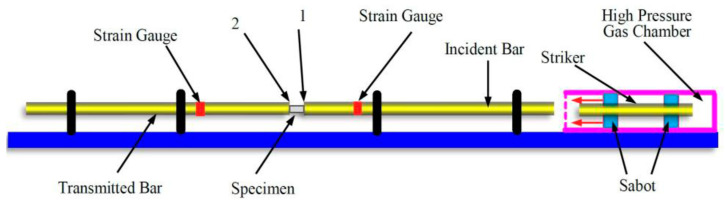
The structural device diagram of SHPB pressure bar.

The structural device diagram of the SHPB pressure bar is shown in Figure 6. The main working principle of the SHPB pressure bar is to use the high-pressure gas in the high-pressure gas chamber to drive the striker (impact bar) to impact the incident rod. According to the strain gauges pasted on the incident bar and the transmission bar, the waveform of the incident wave, the reflected wave and the transmitted wave can be obtained. Based on the one-dimensional stress wave theory, the stress−strain (*σ*−*ε*) relationship of the test sample can be derived.

**Figure 7:**
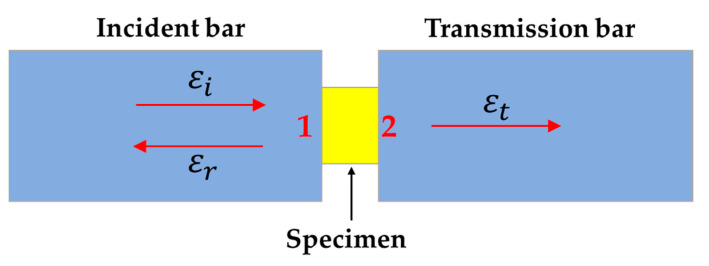
The structural diagram of the local interaction between the test sample and the SHPB pressure bar.

The schematic diagram of the local interaction between the test sample and the SHPB pressure bar is shown in Figure 7. Assuming that the propagation direction of the strain pulse $\varepsilon_{i}$ in the incident bar is positive, the propagation direction of the reflected pulse $\varepsilon_{r}$ is negative, and the propagation direction of the transmitted pulse $\varepsilon_{t}$ is positive. Note that the contact interface between the sample and the incident bar is 1, and the contact interface between the sample and the transmission bar is 2.

Therefore, according to the linear superposition principle of elastic waves, the axial displacement of the contact interface 1 and 2 can be solved, respectively. The specific expressions are as follows:

(6) $$U_{1}\left(t\right)=c_{0}\int_{0}^{t}(\varepsilon_{i}-\varepsilon_{r})dt$$

(7) $$U_{2}\left(t\right)=c_{0}\int_{0}^{t}\varepsilon_{t}dt$$

where $\varepsilon_{i}$, $\varepsilon_{r}$ and $\varepsilon_{t}$ correspond to the strain of incident wave pulse, reflected wave pulse and transmitted wave pulse when propagating independently in the waveguide bar, and $c_{0}$ represents the elastic wave velocity of the waveguide bar. If the original cross-sectional area of the test sample is defined as $A_{0}$, and the original length of the sample is $l_{0}$, then the average strain *ε*(*t*) of the sample can be obtained as follow:

(8) $$\varepsilon (t)=\frac{U_{1}\left(t\right)-U_{2}\left(t\right)}{l_{0}}=\frac{c_{0}}{l_{0}}\int_{0}^{t}(\varepsilon_{i}-\varepsilon_{r}-\varepsilon_{t})dt$$

Taking the derivative of the above equation with respect to time *t*, the strain rate of the test sample can be obtained:

(9) $$\dot{\varepsilon }=\frac{d\varepsilon \left(t\right)}{dt}=\frac{c_{0}}{l_{0}}(\varepsilon_{i}-\varepsilon_{r}-\varepsilon_{t})$$

According to the one-dimensional stress wave theory, the axial forces at contact interface 1 and 2 can be obtained as follows:

(10) $$F_{1}\left(t\right)=AE(\varepsilon_{i}+\varepsilon_{r})$$

(11) $$F_{2}\left(t\right)=AE\varepsilon_{t}$$

Thus, the average stress in the test sample can be obtained as:

(12) $$\sigma \left(t\right)=\frac{F_{1}\left(t\right)+F_{2}\left(t\right)}{2A_{0}}=\frac{AE}{2A_{0}}(\varepsilon_{i}+\varepsilon_{r}+\varepsilon_{t})$$

In the Formulas (10)–(12), *A* represents the cross-sectional area of the waveguide bar, *E* represents the Young’s modulus of the waveguide bar. When the interface 1 and interface 2 of the test sample are in force balance state, there is the following relationship:

(13) $$F_{1}\left(t\right)=F_{2}\left(t\right)$$

Combined with the assumption of uniformity, it can be considered that uniform deformation and uniform force are generated inside the test sample. Therefore, the average stress represents the one-dimensional stress state inside the test sample, and the following relationship can be obtained:

(14) $$\varepsilon_{i}+\varepsilon_{r}=\varepsilon_{t}$$

Substituting the Formula (14) into the Formulas (8), (9) and (12), the following relationship can be further obtained:

(15) $$\dot{\varepsilon }=-\frac{2c_{0}}{l_{0}}\varepsilon_{r}$$

(16) $$\varepsilon \left(t\right)=-\frac{2c_{0}}{l_{0}}\int_{0}^{t}\varepsilon_{r}dt$$

(17) $$\sigma \left(t\right)=\frac{AE}{A_{0}}\varepsilon_{t}$$

The dynamic stress−strain relationship of the sample can be obtained by combining the Formulas (16) and (17). It can be seen that the dynamic stress−strain relationship of the test sample can be determined only by measuring any two waveforms in the incident wave, reflected wave and transmitted wave. Similarly, the stress−strain relationship obtained by the SHPB pressure bar is still the engineering stress−strain relationship. According to the above analysis, the relationship between the true stress−strain and the engineering stress−strain of the material is still shown in the Formula (5).

### 3.2. Sample Preparation and Test Results

**Figure 8:**
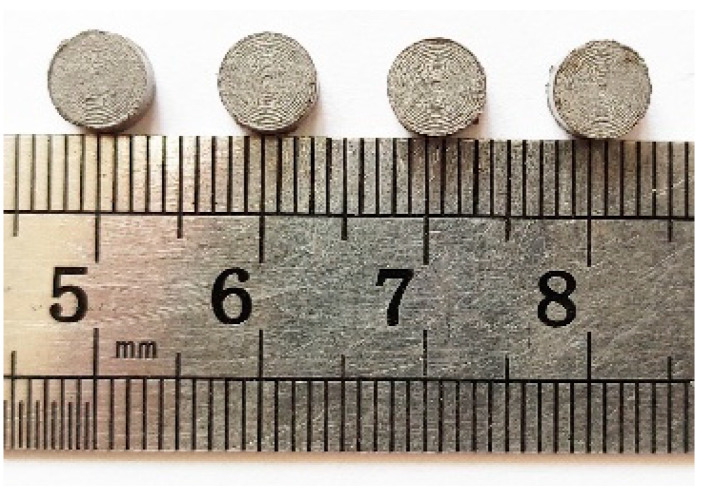
The physical diagram of PTFE/Al/Si samples for dynamic mechanical properties test.

The SHPB pressure bar test was completed in the light gas gun laboratory of the National University of Defense Technology. In the whole loading test device, the bullet and waveguide rod are made of hard aluminum alloy, in which the size of the bullet is ∅ 20 × 300 mm, the size of the incident rod is ∅ 20 × 2800 mm and the transmission rod size is ∅ 20 × 1500 mm. During the test, the bullet velocity is controlled by adjusting the chamber pressure to achieve different loading strain rates. The sample size for testing dynamic mechanical properties of the PTFE/Al/Si reactive materials is ∅ 6 × 3 mm, and its physical diagram is shown in Figure 8.

In order to master the dynamic mechanical properties of PTFE/Al/Si reactive material, the dynamic mechanical responses of PTFE/Al/Si reactive material under different loading strain rates and different test temperatures were studied in this paper.

#### 3.2.1. Test Results under Different Loading Strain Rates

In order to obtain the strain rate effect of PTFE/Al/Si reactive materials, three different loading strain rates (3500 s^−1^, 4500 s^−1^, 5500 s^−1^) were designed for experimental study, and the test temperature remains constant at 25 °C. The test is repeated three times for each loading strain rate, and the size parameters of samples are shown in Table 3.

**Figure 9:**
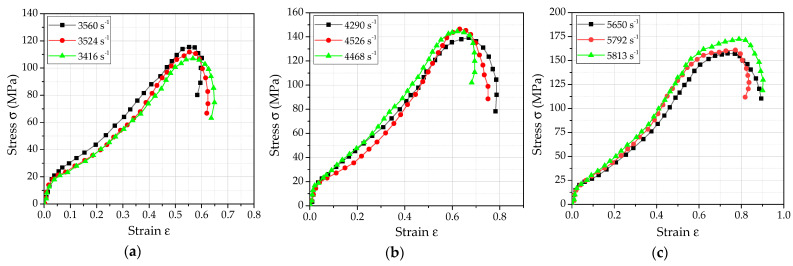
The true stress−strain relationship curve of the PTFE/Al/Si reactive material under different loading strain rates: (**a**) 3500 s^−1^; (**b**) 4500 s^−1^; (**c**) 5500 s^−1^.

By processing the signal collected by the oscilloscope, the true stress−strain relationship curve of the PTFE/Al/Si reactive material under different loading strain rates is shown in Figure 9.

**Figure 10:**
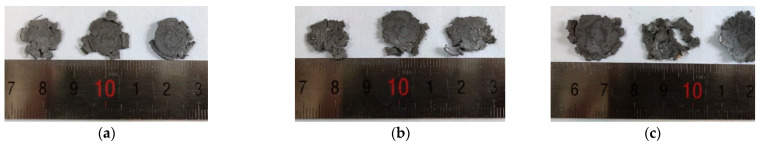
The recycled sample under different loading strain rates: (**a**) 3500 s^−1^; (**b**) 4500 s^−1^; (**c**) 5500 s^−1^.

**Figure 11:**
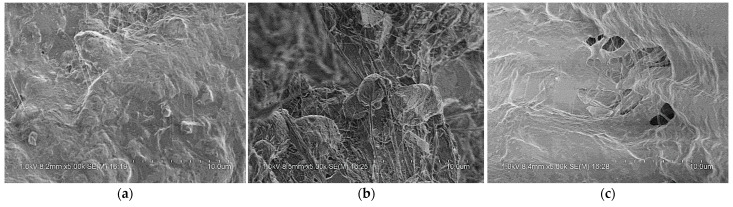
Scanning electron microscope photographs of recycled samples under different loading strain rates: (**a**) 3500 s^−1^; (**b**) 4500 s^−1^; (**c**) 5500 s^−1^.

By analyzing Figure 9, the main dynamic performance parameters of PTFE/Al/Si reactive material at different loading strain rates were obtained as shown in Table 4. In addition, the samples were recycled to obtain the final fracture morphology of the sample under various loading conditions, as shown in Figure 10. In order to observe and analyze the structural failure of samples under different strain rates, the recycled samples were also examined by scanning electron microscope (TESCAN CHINA, Ltd., Shanghai, China), as shown in Figure 11.

**Figure 12:**
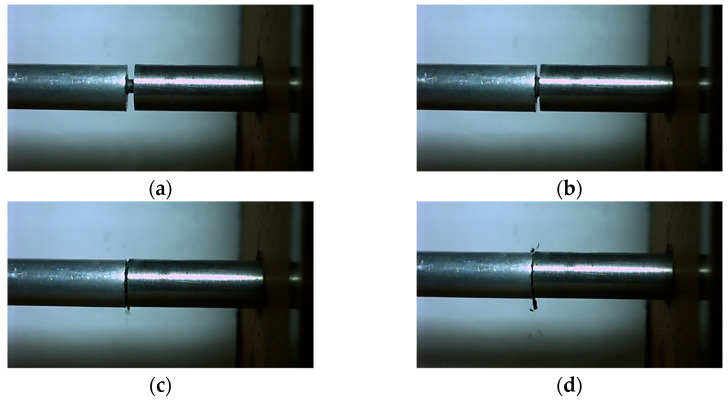
The interaction process between the bars and the sample at different times: (**a**) t = 0 μs; (**b**) t = 333 μs; (**c**) t = 666 μs; (**d**) t = 999 μs.

By analyzing the crushing form of the recycled samples shown in Figure 10, it can be seen that the samples were broken under different loading strain rates, but the sample shows certain plasticity in the crushing process, rather than splashing in the form of powder. In order to better illustrate the crushing process of the specimen under impact, taking the working condition corresponding to the loading strain rate of 4526 s^−1^ as an example, the interaction process between the bars and the sample is shown by high-speed photography, as shown in Figure 12.

#### 3.2.2. Test Results under Different Temperatures

Since the main component of the PTFE/Al/Si reactive material studied in this paper is PTFE, the temperature softening effect of the reactive material needs to be considered. Therefore, four different temperatures (25 °C, 100 °C, 150 °C, 200 °C) were designed to carry out the study at the loading strain rate of 4500 s^−1^, and the size parameters of samples are shown in Table 5. The specific implementation steps of different test temperatures are as follows: firstly, the samples are placed in an adjustable thermostat for heating (considering the heat loss after the sample is taken out, the setting temperature is 1.2 times the actual temperature) until the sample temperature reaches the set temperature. Then, the sample for clamping is quickly taken out, and the loading test is started. It is worth noting that the test results corresponding to 25 °C here are completely consistent with #4, #5 and #6, corresponding to Section 3.2.1. Therefore, the test results corresponding to 25 °C are no longer displayed in this subsection.

**Figure 13:**
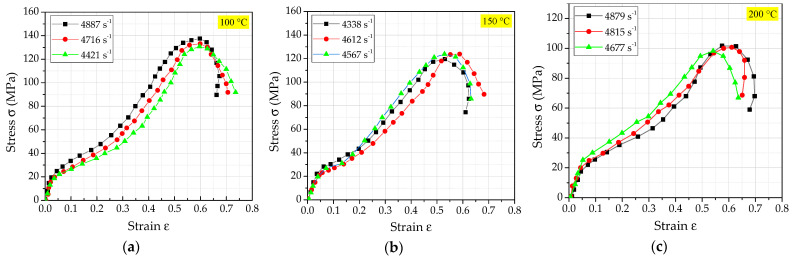
The true stress−strain relationship curve of the PTFE/Al/Si reactive material at different temperatures: (**a**) T = 100 °C; (**b**) T = 150 °C; (**c**) T = 200 °C.

**Figure 14:**
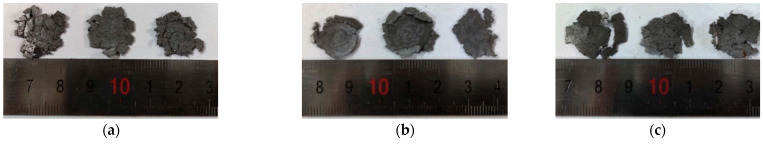
The recycled sample at different temperatures: (**a**) T = 100 °C; (**b**) T = 150 °C; (**c**) T = 200 °C.

**Figure 15:**
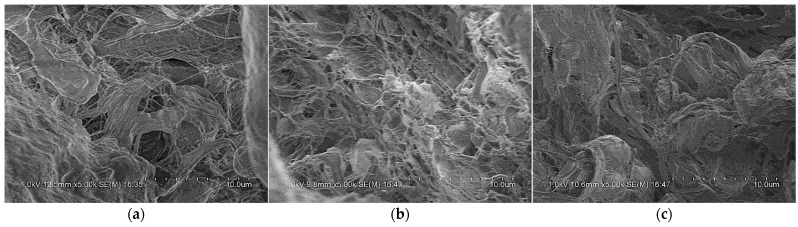
Scanning electron microscope photographs of recycled samples at different temperatures: (**a**) T = 100 °C; (**b**) T = 150 °C; (**c**) T = 200 °C.

Similarly, by processing the signals collected by the oscilloscope, the true stress−strain relationship curves of PTFE/Al/Si reactive materials under different temperature conditions at the loading strain rate of 4500 s^−1^ were obtained as shown in Figure 13. According to the analysis of Figure 13, the main dynamic performance parameters of PTFE/Al/Si reactive materials at different temperature conditions were obtained as shown in Table 6. It can be obviously seen that with the increase of temperature, the corresponding hardening modulus of PTFE/Al/Si reactive materials shows a decreasing trend. The samples after the test at different temperatures were recycled, and the final crushing morphology of the recycled samples under various loading conditions were also obtained, as shown in Figure 14. Similarly, the scanning electron microscope photographs of each recycled sample can be obtained as shown in Figure 15.

## 4. Constitutive Model of the PTFE/Al/Si Reactive Materials

When the material is subjected to external load, the material will produce phenomena such as yield, deformation and fracture. In engineering applications, it is usually expressed by a functional relationship in order to facilitate application. Therefore, the constitutive model, which can characterize the internal characteristics of materials, is produced. The constitutive model of materials can describe the functional relationship between the load and motion or deformation of materials in spatial and temporal coordinates. In essence, the constitutive model is a quantitative mathematical expression of the internal law of deformation and flow of materials after stress, which is usually expressed as the functional relationship of stress, strain and strain rate [26]. Therefore, the constitutive model of materials can reflect the mechanical response behavior of materials under external loads (loading strength, loading rate, etc.).

### 4.1. Selection of Constitutive Model

According to the reactive material formula designed in this paper, the main basic component is PTFE. At present, several common thermo-plastic constitutive models mainly include: the Bodner-Paton model, the Johnson-Cook model, the Follansbee-Kocks model, the Zerorilli-Armstrong model, etc. [27]. The main construction concept of the Bodner-Paton constitutive model is to divide the total strain tensor of the material into an elastic part (described by Hook’s law) and a plastic part (the relationship between the strain rate tensor and J_2_ is constructed according to the dislocation dynamics). However, due to the large number of material parameters required by this model, it is relatively difficult to apply. The main construction concept of the Follansbee-Kocks model is to use the critical stress of the material as an internal variable. The disadvantage of this model is that it also introduces more material parameters and its expression is more complicated. Compared with the previous two models, the specific expressions of the Johnson-Cook model and the Zerrilli-Armstrong model are relatively simple. The common feature of the two models is that the thermal softening parameters, strain hardening parameters and strain rate hardening parameters of materials are introduced. The main construction concept of the Johnson-Cook model is to construct the constitutive relationship of materials based on a large number of experimental data, which can well solve the mechanical response behavior of materials under high strain rate loading, large deformation and high temperature, and is suitable for materials with various crystal structures [28]. The Zerrilli-Armstrong model is mainly applicable to face-centered cubic and body-centered cubic metal materials, and the corresponding expression forms are different with different crystal structures.

Based on research literature, PTFE/Al based reactive materials show a strain rate hardening effect and a strain hardening effect under different loading strain rates. Therefore, this paper will combine the quasi-static and dynamic test data above and refer to the expression of the Johnson-Cook model to construct the constitutive model of the PTFE/Al/Si reactive material.

The Johnson-Cook model was first proposed by Johnson and Cook at the 7th International Ballistic Conference in 1983 [29]. The calculation results obtained based on this model are in good agreement with the experimental results, and the expression form is relatively simple and easy to solve, which makes the Johnson-Cook model widely used at present. At present, the expression form of the Johnson-Cook model is not uniform. This paper refers to the expression in the LS-DYNA keyword manual (*MAT_JOHNSON_COOK). The specific expression form is as follows:

(18) $$\sigma_{y}=\left(A+B{\bar{\varepsilon }}_{p}^{n}\right)\left[1+C\ln\left(\dot{\varepsilon }/{\dot{\varepsilon }}_{0}\right)\right]\left[1-{\left(\frac{T-T_{r}}{T_{m}-T_{r}}\right)}^{m}\right]$$

where *A* is the yield strength, *B* is the strain hardening coefficient, *n* is the strain hardening index, *C* is the strain rate sensitivity coefficient, *m* is the temperature softening coefficient, ${\bar{\varepsilon }}_{p}$ is the equivalent plastic strain, $\dot{\varepsilon }$ is the equivalent strain rate, ${\dot{\varepsilon }}_{0}$ is the reference strain rate, $T_{r}$ is the environmental temperature and $T_{m}$ is the melting temperature of the sample to be tested.

In addition, it can be seen from the expression that the Johnson-Cook constitutive model is a viscoelastic model related to strain rate and temperature. The three terms in the above formula describe the strain hardening effect, strain rate hardening effect and thermal softening effect of the material, respectively. The structure of the entire constitutive model is simple and clear, and the physical meaning of each expression is easy to understand. The constitutive model contains five undetermined parameters: *A*, *B*, *C*, *n* and *m*. These five parameters can be determined by a quasi-static tension compression test, a torsion test and an SHPB test (or Taylor test) at different temperatures and strain rates.

### 4.2. Establishment of Johnson-Cook Constitutive Model Parameters

#### 4.2.1. Strain Hardening Effect

As can be seen from the foregoing, the first term $\left(A+B{\bar{\varepsilon }}_{p}^{n}\right)$ in Formula (18) represents the strain hardening effect of material. In order to obtain the three parameters *A*, *B* and *n* in the Johnson-Cook constitutive model, the stress−strain curves of quasi-static compression tests at room temperature need to be processed. In the quasi-static compression test, the loading speed of the punch is 0.01 mm/s, that is, the loading strain rate $\dot{\varepsilon }$ of the quasi-static test is 0.001 s^−1^. In this paper, the reference strain rate is 0.001 s^−1^, then the second item $\left[1+C\ln\left(\dot{\varepsilon }/{\dot{\varepsilon }}_{0}\right)\right]$ in the Johnson-Cook constitutive model is 1. The ambient temperature of the whole quasi-static compression test is room temperature, and the third item $\left[1-{\left(\frac{T-T_{r}}{T_{m}-T_{r}}\right)}^{m}\right]$ in the Johnson-Cook constitutive model is 1. Thus, the expression of Johnson-Cook constitutive model can be simplified as:

(19) $$\sigma_{y}=\left(A+B{\bar{\varepsilon }}_{p}^{n}\right)$$

**Figure 16:**
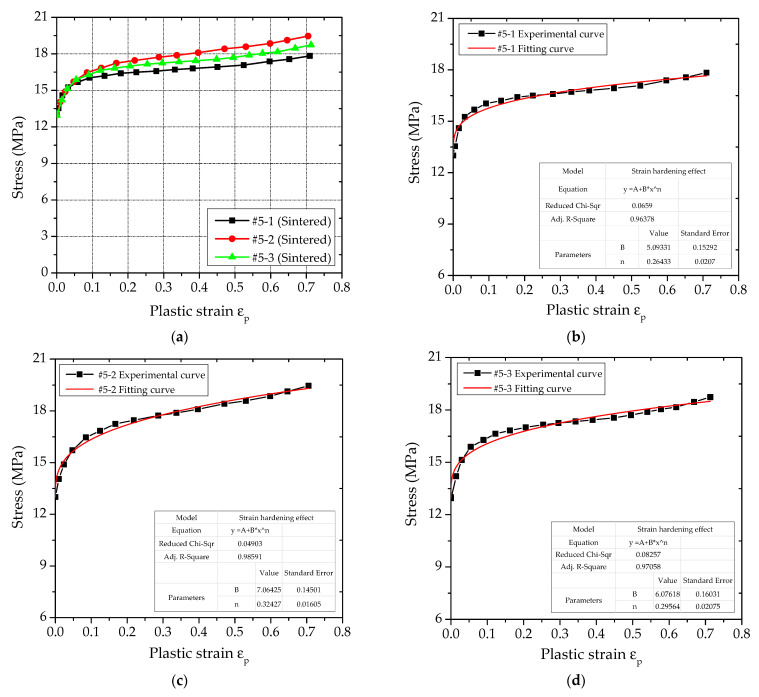
Stress−strain curve and fitting curve of plastic section of PTFE/Al/Si specimen: (**a**) Stress−strain curve of plastic section of PTFE/Al/Si specimen; (**b**) Fitting curve corresponding to # 5-1 working condition; (**c**) Fitting curve corresponding to # 5-2 working condition; (**d**) Fitting curve corresponding to # 5-3 working condition.

In Section 2.2, the true stress−strain curve of the PTFE/Al/Si specimen under quasi-static compression was obtained. In this section, the stress−strain curve of its plastic section was obtained, as shown in Figure 16a. Then, the plastic section data of PTFE/Al/Si specimen were processed and the parameters were fitted, and the fitting curves were obtained as shown in Figure 16b–d. In addition, the fitting values of the parameters included in the strain hardening effect can be obtained as shown in Table 7.

It can be seen from Table 7 that the coefficient of adjusted R^2^ of the three groups of fitting curves are very close to 1, indicating that the fitting effect of the parameters is better. In addition, by analyzing the stress−strain curves of plastic section of three groups of sintered PTFE/Al/Si specimens shown in Figure 16a, it can be found that the curves corresponding to #5-3 are closer to the average values of the three groups of curves. Therefore, the curve parameters fitted by #5-3 are used as the strain hardening effect parameters of the PTFE/Al/Si reactive material. Therefore, the Johnson-Cook constitutive model of PTFE/Al/Si reactive materials only considering strain hardening effect is as follows:

(20) $$\sigma_{y}=13.0+6.076{\bar{\varepsilon }}_{p}^{0.296}$$

#### 4.2.2. Strain Rate Hardening Effect

According to the physical meaning of the Johnson-Cook constitutive model expression, the second item $\left[1+C\ln\left(\dot{\varepsilon }/{\dot{\varepsilon }}_{0}\right)\right]$ reflects the strain rate hardening effect of material. Therefore, if the test is carried out at room temperature, the expression of the Johnson-Cook constitutive model can be reduced to the following format:

(21) $$\sigma_{y}=\left(A+B{\bar{\varepsilon }}_{p}^{n}\right)\left[1+C\ln\left(\dot{\varepsilon }/{\dot{\varepsilon }}_{0}\right)\right]$$

It can be seen from Formula (21) that the strain rate hardening effect term regards the logarithm of stress and strain rate as a linear relationship, but the expression is not necessarily suitable for fluoropolymer-based reactive materials. The literature [28] studied the strain rate hardening effect of PTFE/Al reactive materials. The results show that when the strain rate is low, the flow stress increases rapidly with the increase of strain rate. When the strain rate increases to a certain extent, the strain rate hardening effect of the material decreases, and it is found that the fitting curves corresponding to different plastic strains are approximately parallel, that is, the relationship between strain rate and stress is approximately power exponential. Since the Si content in the PTFE/Al/Si reactive material studied in this paper is relatively small, the material properties are inevitably similar to those of the PTFE/Al reactive material. Therefore, this paper will also use the modified strain rate hardening model, the specific expression of which is as follows:

(22) $$\sigma_{y}=\left(A+B{\bar{\varepsilon }}_{p}^{n}\right){\left(\dot{\varepsilon }/{\dot{\varepsilon }}_{0}\right)}^{\lambda }$$

Since the fitting parameters of *A*, *B* and *n* have been obtained in the previous section according to the quasi-static compression test, in order to obtain the parameter *λ* in the second item it is only necessary to determine the corresponding relationship between plastic strain and stress at different strain rates, and the specific value of parameter *λ* can be obtained by fitting.

In Section 3.2.1, the stress−strain curves of materials at different strain rates were measured. Therefore, based on the above measured data, this section extracts the corresponding stress values when the plastic strain $\varepsilon_{p}$ is 0.25 and 0.35, as shown in Table 8. Taking the loading strain rate $\dot{\varepsilon }$ as the independent variable and the stress $\sigma_{y}$ as the dependent variable, the fitting value of the parameter *λ* can be obtained by fitting the data points.

**Figure 17:**
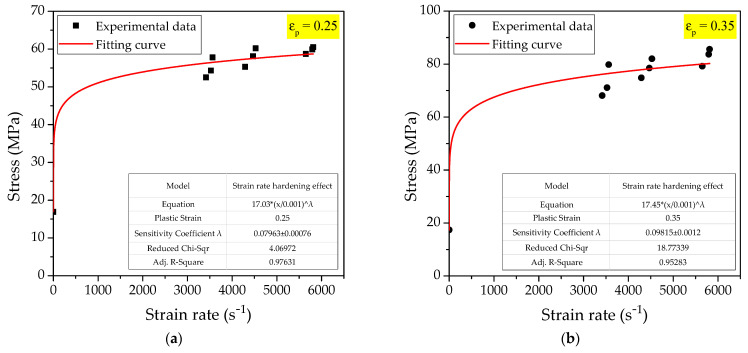
The fitting curve of strain rate hardening effect: (**a**) $\varepsilon_{p}$= 0.25; (**b**) $\varepsilon_{p}$= 0.35.

The data in Table 8 are fitted according to the modified strain rate hardening model, and the corresponding curve fitting relationship with different plastic strains can be obtained, as shown in Figure 17.

Analyzing the fitting curve relationship in Figure 17, it can be seen that the coefficient of adjusted R^2^ when the plastic strain is 0.25 is 0.97631, and the coefficient of adjusted R^2^ when the plastic strain is 0.35 is 0.95283. Obviously, when the plastic strain is 0.25, the corresponding coefficient of determination R^2^ is closer to 1. Therefore, this paper selects the fitting relationship when the plastic strain is 0.25, and the strain rate sensitivity coefficient *λ* is 0.07963. The Johnson-Cook constitutive model of PTFE/Al/Si reactive material considering the strain hardening effect and the strain rate hardening effect is as follows:

(23) $$\sigma_{y}=\left(13.0+6.076{\bar{\varepsilon }}_{p}^{0.296}\right){\left(\dot{\varepsilon }/{\dot{\varepsilon }}_{0}\right)}^{0.07963}$$

#### 4.2.3. Thermal Softening Effect

Through the above analysis of the strain hardening effect and the strain rate hardening effect, the parameters *A*, *B*, *n* and *λ* of the Johnson-Cook model of PTFE/Al/Si reactive material have been determined. In the Johnson-Cook constitutive model, only the temperature softening coefficient *m* in the third term has not been determined. According to the dynamic stress−strain curves of PTFE/Al/Si reactive materials measured at different temperatures when the loading strain rate is 4500 s^−^^1^, the stresses corresponding to different temperatures when the plastic strain $\varepsilon_{p}$ is 0.25 and 0.35 were obtained, as shown in Table 9.

**Figure 18:**
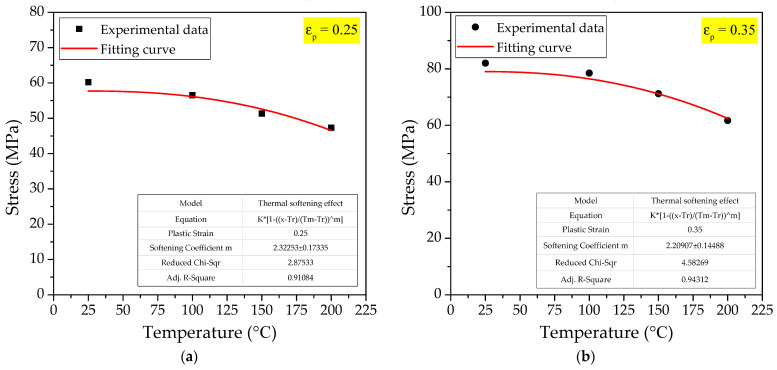
The fitting curve of thermal softening effect: (**a**) $\varepsilon_{p}$= 0.25; (**b**) $\varepsilon_{p}$= 0.35.

Similarly, the fitting curves of the thermal softening effect corresponding to different plastic strain conditions can be obtained by fitting the stress-temperature relationship under different temperature conditions in Table 9, as shown in Figure 18.

In Figure 17, it can also be seen that the coefficient of adjusted R^2^ when the plastic strain is 0.25 is 0.91084, and the coefficient of adjusted R^2^ when the plastic strain is 0.35 is 0.94312. Obviously, when the plastic strain is 0.35, the corresponding coefficient of determination R^2^ is closer to 1. Therefore, this paper selects the fitting relationship when the plastic strain is 0.35 and the thermal softening coefficient *m* is 2.20907.

The five main control parameters *A*, *B*, *C*, *n* and *m* of the Johnson-Cook constitutive model of PTFE/Al/Si active materials have been fully obtained. Therefore, the Johnson-Cook constitutive model expression of the PTFE/Al/Si reactive material considering the strain hardening effect, strain rate hardening effect and thermal softening effect can be obtained as follows:

(24) $$\sigma_{y}=\left(13.0+6.076{\bar{\varepsilon }}_{p}^{0.296}\right){\left(\dot{\varepsilon }/{\dot{\varepsilon }}_{0}\right)}^{0.07963}\left[1-{\left(\frac{T-T_{r}}{T_{m}-T_{r}}\right)}^{2.20907}\right]$$

## 5. Conclusions

Under high-speed dynamic loading, the reactive material will undergo intense and rapid combustion or detonation-like reactions, accompanied by the release of a large amount of chemical energy. In the early stage, the author’s team carried out qualitative and quantitative tests on the energy release ability for different types of reactive material formulations, and finally selected PTFE/Al/Si as the reactive material formulation with the best energy release ability. After determining the formula of the reactive material, the quasi-static mechanical properties test and dynamic mechanical properties test of PTFE/Al/Si reactive material were carried out, and the basic mechanical properties parameters of this new reactive material formula were obtained. In order to carry out the subsequent numerical simulation, the Johnson-Cook constitutive model of PTFE/Al/Si reactive material was constructed according to the measured mechanical property parameters. Finally, the Johnson-Cook constitutive model expression of the PTFE/Al/Si reactive material considering the strain hardening effect, the strain rate hardening effect and the thermal softening effect was obtained as follows:

(25) $$\sigma_{y}=\left(13.0+5.949{\bar{\varepsilon }}_{p}^{0.289}\right){\left(\dot{\varepsilon }/{\dot{\varepsilon }}_{0}\right)}^{0.07963}\left[1-{\left(\frac{T-T_{r}}{T_{m}-T_{r}}\right)}^{2.20907}\right]$$

In the next step, we will carry out the numerical simulation test under high strain rate loading based on the constitutive model of PTFE/Al/Si constructed in this paper, so as to further study the energy release mechanism of PTFE/Al/Si reactive material from the macro and micro perspectives, and further guide the design and application of new reactive material formulations.

## Figures and Tables

**Table 1:** Structural parameters of PTFE/Al/Si reactive material samples.

No.	Diameter (mm)	Height (mm)	Mass (g)
#5-1	9.92	10.02	1.736
#5-2	9.92	9.98	1.735
#5-3	9.92	10.01	1.736

**Table 2:** Main material parameters of the PTFE/Al/Si reactive material.

No.	Elastic Modulus *E* (MPa)	Hardening Modulus *E*_s_ (MPa)	Nominal Yield Strength *σ*_s_ (MPa)	Yield Strain *ε*_s_
#5-1	387.2	2.7	12.9	0.0546
#5-2	375.9	3.8	12.9	0.0556
#5-3	369.6	3.0	13.0	0.0526

**Table 3:** The size parameters of samples under different loading strain rates.

No.	Mass (g)	Diameter (mm)	Thickness (mm)	Loading Strain Rates (s^−1^)
#1	0.186	6.04	3.10	3500
#2	0.181	6.04	3.08	
#3	0.188	6.04	3.10	
#4	0.204	6.04	3.11	4500
#5	0.196	6.04	3.05	
#6	0.197	6.04	3.06	
#7	0.196	6.04	3.02	5500
#8	0.189	6.04	3.06	
#9	0.195	6.04	2.92	

**Table 4:** The main dynamic performance parameters of PTFE/Al/Si reactive material at different loading strain rates.

No.	Loading Strain Rates (s^−1^)	Hardening Modulus (MPa)	Yield Strength (MPa)	Critical Strain
#1	3416	174	17.9	0.5523
#2	3524	180	18.4	0.5533
#3	3560	185	18.2	0.5706
#4	4290	204	18.5	0.6593
#5	4468	206	18.8	0.6210
#6	4526	209	19.2	0.6312
#7	5650	211	19.1	0.7348
#8	5792	219	19.5	0.7519
#9	5813	227	20.1	0.7880

**Table 5:** The size parameters of samples at different temperatures.

No.	Mass (g)	Diameter (mm)	Thickness (mm)	Temperature (°C)
#10	0.191	6.04	3.08	100
#11	0.190	6.04	3.11	
#12	0.189	6.04	3.10	
#13	0.189	6.04	3.07	150
#14	0.195	6.04	3.08	
#15	0.187	6.04	3.05	
#16	0.184	6.04	3.03	200
#17	0.187	6.04	3.08	
#18	0.185	6.04	3.04	

**Table 6:** The main dynamic performance parameters of PTFE/Al/Si reactive material at different temperatures.

Temperature (°C)	Loading Strain Rates (s^−1^)	Hardening Modulus (MPa)	Yield Strength (MPa)	Critical Strain
100	4421	197	16.8	0.5963
	4716	201	17.5	0.5869
	4887	206	18.2	0.5974
150	4338	179	18.1	0.5304
	4567	181	18.3	0.5418
	4612	185	17.9	0.5867
200	4677	165	16.8	0.5412
	4815	169	17.2	0.6011
	4879	173	17.5	0.6089

**Table 7:** Fitting parameters of the strain hardening effect.

No.	*A*	*B*	*n*	Adjusted R^2^
#5-1	13.0	5.093	0.264	0.96378
#5-2	13.0	7.064	0.324	0.98591
#5-3	13.0	6.076	0.296	0.97085

**Table 8:** Stresses corresponding to different plastic strains under different strain rates. (25 °C).

Loading Strain Rate $\dot{\varepsilon }$ (s^−1^)	$\mathrm{Stress}\;\sigma_{y}$$\;(\varepsilon_{p}\;=\;0.25)$ (MPa)	$\mathrm{Stress}\;\sigma_{y}$$\;(\varepsilon_{p}\;=\;0.35)$ (MPa)
0.001	16.9	17.4
3416	52.5	68.1
3524	54.3	71.1
3560	57.8	79.8
4290	55.3	74.8
4468	58.1	78.5
4526	60.2	82.0
5650	58.7	79.2
5792	59.9	83.7
5813	60.5	85.6

**Table 9:** Stresses corresponding to different plastic strains under different temperatures. (4500 s^−1^).

Temperature (°C)	$\mathrm{Stress}\;\sigma_{y}$$\;(\varepsilon_{p}\;=\;0.25)$ (MPa)	$\mathrm{Stress}\;\sigma_{y}$$\;(\varepsilon_{p}\;=\;0.35)$ (MPa)
25	60.2	82.0
100	56.5	78.5
150	51.3	71.2
200	47.3	61.7
